# Linguistic and non-linguistic non-adjacent dependency learning in early development

**DOI:** 10.1016/j.dcn.2020.100819

**Published:** 2020-07-04

**Authors:** Anne van der Kant, Claudia Männel, Mariella Paul, Angela D. Friederici, Barbara Höhle, Isabell Wartenburger

**Affiliations:** a Cognitive Sciences, Department Linguistics, University of Potsdam, Germany; b Max Planck Institute for Human Cognitive and Brain Sciences, Department of Neuropsychology, Leipzig, Germany; c Department of Audiology and Phoniatrics, Charité – Universitätsmedizin Berlin, Germany; d Berlin School of Mind and Brain, Humboldt-Universität zu Berlin, Germany

**Keywords:** Non-adjacent dependencies, Associative learning, Infants, Functional near-infrared spectroscopy, Development

## Abstract

Non-adjacent dependencies (NADs) are important building blocks for language and extracting them from the input is a fundamental part of language acquisition. Prior event-related potential (ERP) studies revealed changes in the neural signature of NAD learning between infancy and adulthood, suggesting a developmental shift in the learning route for NADs. The present study aimed to specify which brain regions are involved in this developmental shift and whether this shift extends to NAD learning in the non-linguistic domain. In two experiments, 2- and 3-year-old German-learning children were familiarized with either Italian sentences or tone sequences containing NADs and subsequently tested with NAD violations, while functional near-infrared spectroscopy (fNIRS) data were recorded. Results showed increased hemodynamic responses related to the detection of linguistic NAD violations in the left temporal, inferior frontal, and parietal regions in 2-year-old children, but not in 3-year-old children. A different developmental trajectory was found for non-linguistic NADs, where 3-year-old, but not 2-year-old children showed evidence for the detection of non-linguistic NAD violations. These results confirm a developmental shift in the NAD learning route and point to distinct mechanisms underlying NAD learning in the linguistic and the non-linguistic domain.

## Highlights

• Non-adjacent dependency learning shows a developmental shift during the third year of life.
• 2-year-old children can learn non-adjacent dependencies in linguistic material from passive listening, while 3-year-old children cannot.
• Non-adjacent dependency learning in 2-year-olds is subserved by left-hemisphere temporal, inferior frontal and parietal brain regions.
• The developmental trajectory for non-adjacent dependency learning differs between the linguistic and the non-linguistic domain.

## 1 Introduction

### 1.1 Adjacent and non-adjacent dependencies

To acquire their native language, infants must learn to understand the rule-based relations between individual words, which make up the syntax of that language. Many of these relations exist between elements that are adjacent in the speech input (such as determiners and nouns). These *adjacent dependencies* are readily acquired by very young infants (Gómez and Maye, 2005; Teinonen et al., 2009) as well as adults (e.g., Reber, 1967). However, due to its hierarchical structure, human language also displays rule-based relations between *non-adjacent* elements, which are separated by intervening elements. These relations, known as *non-adjacent dependencies* (NADs), include for example the relation between the auxiliary and the verb suffix in the sentence *“Mary is singing”*. In natural language, NADs play a pivotal role in understanding and building complex syntactic structure, and the ability to extract them is an important milestone in language acquisition. In the non-linguistic domain, understanding hierarchical relationships also requires NAD learning, such as in music and in action sequences (for a review, see Fitch and Martins, 2014). In the following paragraphs, we will outline the current knowledge of NAD learning in the linguistic and non-linguistic domain and its neural basis.

### 1.2 Development of NAD learning in the linguistic domain

The complex nature of linguistic NADs as the building blocks of complex syntax seems to be reflected in the developmental trajectory of NAD learning. For example, previous behavioral research has suggested that the ability to extract NADs from linguistic input develops later than the ability to extract adjacent dependencies (Gómez, 2002; Höhle et al., 2006; Santelmann and Jusczyk, 1998). While NADs between identical syllables (e.g., “nu-fe-nu”) are readily identified from the age of 7 months (Gervain and Werker, 2013; Marcus et al., 1999), extracting these dependencies could depend on repetition detection.

NADs between non-identical elements, however, are both more likely to signify grammatical relations in natural language and require learning the arbitrary relation between the dependent elements (Wilson et al., 2018). At the behavioral level, there is evidence from different languages that NAD learning can be seen from the age of 12 months onwards. Yet, the success of NAD learning depends on a considerable number of factors, including input variability and listeners’ attention. For example, increased variability in the intervening word was shown to improve NAD learning (Gómez, 2002), while attention during NAD learning modulates the kind of knowledge that is acquired and retained (López-Barroso et al., 2016). Accordingly, in natural language learning studies, the exact age at which NADs were shown to be learned depends on the material used, the task design, and/or the specific languages that were tested. Specifically, NAD learning in syllable triplets from a segmented speech stream has been shown in 12-month-old infants (Marchetto and Bonatti, 2013, 2015) and from a continuous speech stream in 18-month-olds (Marchetto and Bonatti, 2013). At around 15 months of age, infants are able to track NADs between the first and last word in 3-word sentences (Gómez and Maye, 2005), while at 12 months they can only do so if they learn to track adjacent dependencies first (Lany and Gómez, 2008). When adapted to Dutch phonotactics, these NADs were learned by 18-month-old Dutch-learning infants, but not generalized to new contexts (Grama and Wijnen, 2018). By 18 months, American English-learning infants recognize the learned *“is_ing”*-dependency (Santelmann and Jusczyk, 1998). French-learning infants detect subject-verb agreement (e.g., the de *“les_ils”-*dependency in *“les garçons ils arrivent”* (where “-ent” is silent), which translates to “the boys are coming”) marking from the age of 14 months, but cycled through different preferences until they were 24 months old (Culbertson et al., 2016). By the age of 17 months, French-learning infants can track NADs across phonological phrase boundaries (van Heugten and Shi, 2010), and by 18 and 24 months they can detect number marking, which is highly irregular in French (Nazzi et al., 2011). In German, which has a relatively free word order, 19-month-old infants were able to recognize NADs over a maximum of two intervening syllables (Höhle et al., 2006). Compared to behavioral studies, neurophysiological measures have provided evidence for even earlier NAD learning, namely in 3−8-month-old German-learning and French-learning infants (Friederici et al., 2011; Kabdebon et al., 2015; Mueller et al., 2012). These findings will be further outlined in Section 1.4 below.

Comparable to infant studies, behavioral studies in adults have also revealed successful NAD learning when adults were tested under the same passive listening conditions (Frost and Monaghan, 2016; Gómez, 2002; Onnis et al., 2004; Wang et al., 2019). The majority of studies in adults, however, involves metalinguistic components from active listening paradigms, with either a 2AFC task (e.g., Frost and Monaghan, 2016), a familiarity judgment task (Wang et al., 2019), or a grammatical judgment task (e.g., Gómez, 2002; Onnis et al., 2004). Thus, it remains unclear whether behavioral studies assessing NAD learning in adults tap into the same learning mechanisms as infant behavioral NAD learning studies. Nevertheless, there are some similarities across development regarding the factors influencing NAD learning. In infants and adults alike, linguistic NAD learning is enhanced by phonological (Frost and Monaghan, 2016; Newport and Aslin, 2004) and prosodic cues (Grama et al., 2016), very low and very high variability in the intervening, between-dependency element (e.g., Gómez, 2002; Onnis et al., 2004), and attention directed at the dependent elements (e.g., Pacton and Perruchet, 2008).

Taken together, the results of the reviewed studies suggest that the age at which infants show successful NAD learning is influenced by the type of NAD in a given language, the presence of cues aiding NAD learning, and also depends on the method with which learning is measured (e.g., behavioral versus neurophysiological methods). Moreover, behavioral NAD learning success in adults might be influenced by metalinguistic processing required at test. For a comprehensive review on different NAD varieties and the influence of different cues, see Wilson et al. (2018).

### 1.3 Domain-generality of NAD learning

The literature on adult NAD learning shows parallels between linguistic and non-linguistic NAD learning, which suggests some influence of domain-general learning mechanisms. For example, perceptual cues were shown to enhance behavioral NAD learning in the non-linguistic domain in adults for sequences of flute and violin timbres (Creel et al., 2004), sequences of musical tones (Endress, 2010), and sequences of Macintosh alert sounds (Gebhart et al., 2009), which is in line with the findings on linguistic NAD learning discussed above. Furthermore, guiding attention to dependent elements (e.g., by re-typing sequences) improves both linguistic and non-linguistic NAD learning (Pacton et al., 2015 - sequences of digits and syllable sequences; Pacton and Perruchet, 2008 - sequences of digits; Uddén et al., 2017 - sequences of letters). At the neural level, processing of learned NADs in both syllable sequences (Friederici et al., 2006) and sequences of letters (Uddén et al., 2017), involves the left Broca’s area in adults, while processing learned NADs in music primarily recruits the right inferior frontal gyrus, specifically the right homologue of Broca’s area (Cheung et al., 2018). Thus, adults seem to show some parallels but also differences between NAD processing in the linguistic and the non-linguistic auditory domain.

Animal studies provide another perspective on the possible domain-generality of NAD learning. When passively exposed to NADs in syllable triplets in an oddball paradigm, macaques showed ERP patterns that were comparable to human infants, but not to human adults (Milne et al., 2016). These results suggest that NAD learning in infants and adults is rooted in different neural mechanisms, pointing to a qualitative change over development which might be related to the recruitment of different pathways of brain connectivity (Milne et al., 2016), while there might be only quantitative differences in the mechanisms for NAD learning between human infants and non-human primates (Mueller et al., 2018)

If domain-general learning mechanisms guide NAD learning in children, we expect linguistic and non-linguistic NAD learning to show similar developmental trajectories. Since no studies to date report on NAD learning outside the linguistic domain in children under the age of 3 years, we set out to investigate whether the developmental trajectory for non-linguistic NAD learning mirrors that for linguistic NAD learning, that is, we ask whether potential changes in NAD learning capacity between the ages of 2 and 3 years are found both in the linguistic and in the non-linguistic domain. In addition, we aim to reveal whether NAD learning inside and outside the linguistic domain is subserved by the same neural mechanisms in childhood. In the next paragraph we will briefly review what is known about the brain regions involved in NAD learning.

### 1.4 Neural basis of NAD learning

A growing body of fMRI literature reports on the neural basis of NAD learning in adulthood. Specifically Broca’s area was shown to play an important role in the detection of NAD violations after NAD learning, both in studies where participants were informed that they were learning a grammar (i.e., explicit learning that is controlled by top-down processes) (Bahlmann et al., 2008; Friederici et al., 2006), as well as in implicit NAD learning, where participants were told to re-type letter strings during learning (i.e., implicit learning based on bottom-up information) (Uddén et al., 2017). The involvement of Broca’s area in implicit and explicit NAD learning in adults suggests that adult NAD learning is subserved by the same learning mechanisms that underlie processing of complex hierarchical linguistic structure (see Makuuchi et al., 2009).

To date, only a limited number of studies have addressed the neural basis of NAD learning in infants or children. First, Friederici et al. (2011) showed that 4-month-old German-learning infants are able to learn NADs from passive auditory exposure to short sentences in a non-native language (i.e., Italian), reflected by a late positivity in the ERP signal in response to NAD violations. The authors interpreted the late positive ERP signal as a memory-based deviance effect, which might be a precursor to grammatical rule learning. Second, in an oddball paradigm, 3-month-old infants show a mismatch response (MMR) (Mueller et al., 2012), while 2-year-old children show a (related) Late Difference Response (LDR) when detecting NAD violations in syllable triplets (Mueller et al., 2019). Finally, Kabdebon and colleagues demonstrated NAD learning in syllable triplets from a continuous speech stream at 8 months of age (Kabdebon et al., 2015). Both for Italian sentences and syllable triplets in an oddball paradigm, adults’ ERP patterns differed from those found in infants. Adults showed an N400, a negativity in the ERP signal related to lexical processing, when learning NADs from Italian sentences by mere exposure (Friederici et al., 2013; Mueller et al., 2009) or an N400 in combination with an attention-modulated P2 component when learning NADs in trisyllabic strings under passive listening (de Diego-Balaguer et al., 2015; de Diego Balaguer et al., 2007). However, when the prefrontal cortex (PFC) was downregulated by means of Transcranial Direct Current Stimulation during the passive exposure preceding the NAD test, adults showed a positive ERP response during grammatical judgment, which was comparable to the infants’ responses, which might be explained by the involvement of an associative learning mechanism (Friederici et al., 2013). A positivity was also found in adults when the exposure phase was lengthened considerably (Citron et al., 2011). Furthermore, adults learned NADs from syllable triplets in an oddball paradigm (as shown by an MMR in combination with a P3 component) when they were given an explicit instruction to detect violations (Mueller et al., 2012).

The literature reviewed above suggests that the neural mechanisms for NAD learning undergo a developmental shift, where the learning route might change from more implicit, associative learning in infants (effective under passive listening) to primarily controlled, explicit learning in adults (effective when using a task or directing attention). Since Mueller and colleagues found that already at 4 years of age, only a subset of children showed neural evidence of NAD learning under passive listening (Mueller et al., 2019), this shift appears to take place between the ages of 2 and 4 years. Based on the studies reviewed above Skeide and Friederici (2016) proposed a developmental shift from associative learning in infants, mainly supported by temporal brain regions, to controlled learning in adults, primarily subserved by prefrontal brain regions. This shift was proposed to be driven by maturation of the PFC and its functional and white matter connections to the temporal cortex and is expected to occur between the ages of 2 and 4 years (Friederici et al., 2011; Mueller et al., 2019). To date, there is –to the best of our knowledge– no empirical evidence as to which underlying brain regions are involved in infant NAD learning under passive listening conditions (which might lead to associative learning). In the present study, we use fNIRS to assess the brain regions underlying the detection of NAD violations after passive listening in 2- and 3-year-old children, the age range where the neural signature of NAD learning under passive listening is expected to change.

### 1.5 Present study

In addition to replicating the developmental shift in NAD learning shown by Mueller et al. (2019), the present study aims to answer two questions regarding NAD learning in infancy: 1) Which underlying brain regions are involved in processing NADs learned from passive listening in 2- and 3-year-olds? 2) How does the developmental trajectory of NAD learning and its neural basis compare across linguistic and non-linguistic NAD learning? We aim to answer these questions by passively exposing 2- and 3-year-old children to NADs embedded in Italian sentences and pure tone sequences and subsequently using fNIRS to measure the brain’s response to violations of these NADs. In line with earlier work on NAD learning in the non-linguistic domain (e.g., Creel et al., 2004; Endress, 2010; Winkler et al., 2018), non-linguistic NAD sequences were constructed from pure tones.

Based on the literature reviewed above, a change in the neural mechanisms supporting NAD learning under passive listening conditions is expected to occur during the third year of life. Therefore, we expect children to show different neural signatures for NAD processing after passive exposure at 2 years compared to 3 years of age, possibly resulting in a better neural discrimination between familiarized NADs and NAD violations in 2-year-olds compared to 3-year-olds after passive listening. Specifically, we expect the left-hemispheric fronto-temporal language network to be involved in processing linguistic NAD violations. Since adults were shown to learn NADs equally well in the linguistic and in the non-linguistic domain, we expect a similar developmental trajectory for learning in both domains. However, based on adult studies on NAD processing in music (Cheung et al., 2018), we expect processing of violations in non-linguistic NADs to be less left-lateralized compared to linguistic NAD processing.

## 2 Materials and methods

### 2.1 Participants

A total of 96 typically-developing monolingual German-speaking children participated in this study, of which 51 children were 2 years old. Fifty-nine children participated in the linguistic and non-linguistic experiments on different days (35 2-year-olds; 24 3-year-olds), whereas 37 children participated only in the linguistic (8 2-year-olds; 12 3-year-olds) or the non-linguistic experiment (8 2-year-olds; 9 3-year-olds). Due to cancellations of appointments and poor compliance in some sessions, it was not possible to test all children in both experiments, resulting in a data set with both within-subject and between-subject data. Twelve additional 2-year-old children and one 3-year-old child came to the lab but could not be tested, because of non-compliance. The dataset of one additional 2-year-old child was excluded, as a history of hearing problems was reported.

For 32 children, data from both experiments could be included (17 2-year-olds; 15 3-year-olds) after exclusion of movement artifacts and sessions with too few remaining trials (see Section 2.4 for inclusion criteria). For another 36 children, only the linguistic (8 2-year-olds; 10 3-year-olds) or non-linguistic (8 2-year-olds; 10 3-year-olds) data were included. This resulted in a data set with 25 children per age group (2 and 3 years) for both the linguistic (2 years: mean age 25;8 months;days, range 24;1–26;19, 10 boys, 3 years: mean age 37;6 months;days, range 36;2-38;11, 11 boys) and the non-linguistic experiment (2 years: mean age: 24;30 months;days, range: 23;13-26;7, 10 boys) 3 years: mean age: 37;1 months;days, range: 35;10–38;22, 12 boys). Since less than half of the children provided sufficient quality data for both the linguistic and non-linguistic experiments, these were analyzed separately (see 2.5 for details). No history of neurocognitive impairment, language disorders, or hearing impairment was reported for the included children, nor did they have experience with Italian. Children were recruited through direct mail via the BabyLab at the University of Potsdam, with the contact data provided by the Potsdam city council. The study was carried out in accordance with the “Code of Ethics of the World Medical Association (Declaration of Helsinki) for experiments involving humans”. Written informed consent was obtained from the caregivers of all participating children prior to the procedure. Caregivers received a compensation of €7,50 for their participation in this study. The experimental procedure for this study was approved by the Ethics Committee of the University of Potsdam (approval number 17/2015).

### 2.2 Stimuli

**Fig. 1:**
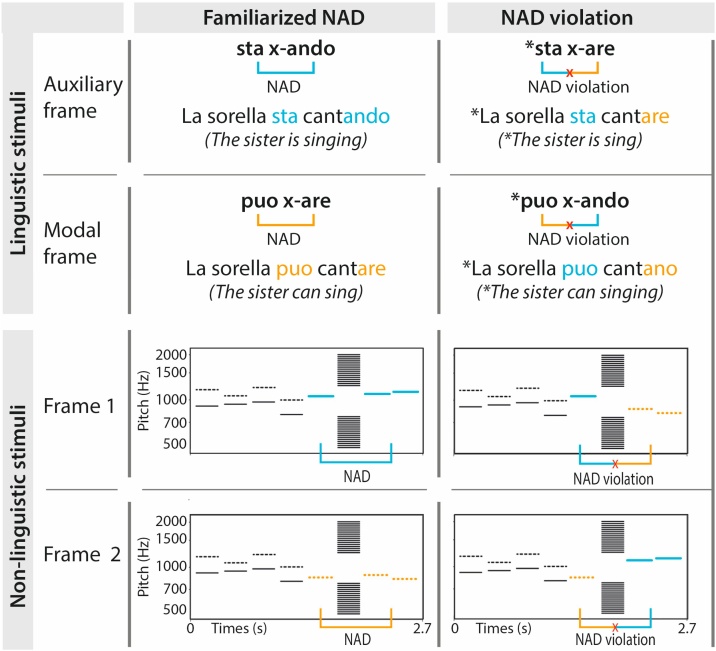
Linguistic (top) and non-linguistic (bottom) stimuli containing non-adjacent dependencies (NADs). Linguistic stimuli comprise a miniature version of Italian, where an auxiliary frame and a modal frame are combined with 32 different verb stems, while non-linguistic stimuli consist of pure tone sequences where every syllable position from the linguistic stimuli is replaced by a pure tone. Due to counterbalancing, this figure represents the familiarized NADs and NAD violations for half of the participants. The other half was familiarized with sta x-are and puo x-ando and were presented with sta x-ando and puo x-are as NAD violations in the test phase (the same was true for the non-linguistic stimuli).

The linguistic material used in this experiment comprised a mini-version of Italian previously used in infant and adult ERP studies (e.g., Friederici et al., 2011, 2013). The material consisted of short sentences containing a noun phrase (*Il fratello/La sorella; the brother/the sister*) followed by an auxiliary or modal verb (*sta/puó; is/can*) and a verb stem (32 tokens) with suffix (*ando/are*). An NAD existed between the auxiliary or modal verb and the verb suffix, according to the pattern depicted in Fig. 1. Ungrammatical test stimuli were generated by combining the auxiliary/modal verb with the alternative suffix. This resulted in a total of 128 grammatical and 128 ungrammatical sentences. Stimuli had a mean length of 2.58 s (see Friederici et al., 2011, for an in-depth description of the stimuli). Although we used natural language, the stimulus sentences were unfamiliar to the children, as were the underlying grammatical dependencies. Any knowledge about the dependency was therefore assumed to originate from the exposure the children received in the lab. Additionally, the particular auxiliary/modal-suffix combination was counterbalanced between participants to control for any intrinsic biases toward the native Italian dependencies. Half of the participants were thus exposed to correct Italian sentences (i.e., *sta*-x-*ando* and *puó-x-are*) during the familiarization and tested on the incorrect sentences, while the other half of children were familiarized with incorrect Italian sentences (i.e., *sta*-x-*are* and *puó-x-ando*) and tested with correct sentences. Throughout this paper, we will refer to the NADs that participants heard during the familiarization phase as “familiarized NADs” and to the non-familiarized NADs (opposite combinations of auxiliary/modal verb and suffix) as “NAD violations”, irrespective of their grammaticality in Italian.

Non-linguistic stimuli were constructed from pure tones using Praat software (Boersma and Weenink, 2016). Since (native-language) phonological knowledge was shown to influence the processing of complex non-linguistic sounds (Azadpour and Balaban, 2008; Berent et al., 2010) we used pure tones to minimize this influence. A full set of 46 pure tones was generated in Praat, from which 8-tone sequences were generated by substituting each syllable position in the linguistic stimuli with a pure tone. Tones were generated within the 500−2000 Hz frequency range, a range which covers the most important frequencies in human speech. Tones were generated in equal steps on a logarithmic scale to ensure they are perceived as equidistant in pitch (Greenwood, 1961). The frequencies of the tones comprising the NADs were 1047 Hz for “*sta”*, 870 Hz for “*puó”*, 1114 Hz for the first syllable and 1149 Hz for the second syllable of “-*ando”*, and 898 Hz for the first syllable and 844 Hz for the second syllable of “-*are*”. The carrier sentences “*Il fratello”* and *“la sorella”* also comprised tones in the 818−1222 Hz frequency range. Tones occurring at the original position of the verb stems ranged from 500 Hz to 794 Hz and from 1260 Hz to 2000 Hz (see Fig. 1). Note that the two tones which represent the two syllables of the suffix (tone 7 and 8) are close in frequency, as are the tone which represents the auxiliary / modal verb (tone 5) and tone 7 in the familiarized NADs shown in Fig. 1. However, since the relation between tone 5 and the suffix (tones 7 and 8) was counterbalanced between participants, we do not expect this to influence our results. Transitional probabilities were identical in the linguistic and non-linguistic stimulus sets. All pure tones were of the same duration (270 ms), which corresponded to the mean duration of syllables in the linguistic stimuli. The overall duration of tone sequences (2580 ms) and the duration of the pauses between tones (60 ms) were also derived from the respective means of the linguistic stimulus set.

### 2.3 Procedure

The experimental procedure consisted of a 5-minute-long familiarization phase followed by a 20-minute-long test phase. In adults, this design was shown to enhance NAD learning compared to a design which alternates shorter learning and test phases (Citron et al., 2011). Detection of NAD violations after learning was assessed using fNIRS, a non-invasive technique that uses near-infrared light to measure blood oxygenation changes, which reflect changes in neural activity. During familiarization, the child was presented with 100 sentences containing either the *sta x-ando* and *puó x-are* or the *sta x-are* and *puó x-ando* NADs (or the tone-sequence equivalents) and no fNIRS data were recorded.

**Fig. 2:**
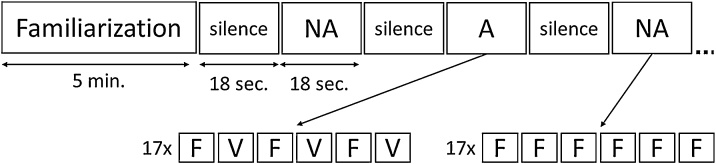
Alternating-non-alternating paradigm for presentation of the stimuli during fNIRS data acquisition. NA: non-alternating, A: Alternating, F: familiarized NAD, V: NAD violation.

Immediately after familiarization and unbeknownst to the participants, the test phase started, during which fNIRS data were recorded from the child’s frontal, inferior frontal, temporal and parietal brain regions. Here, an alternating-non-alternating-paradigm (Gervain et al., 2012) was employed to test fine-grained neural discrimination of familiarized NADs vs. NAD violations. That is, we presented 17 non-alternating (NA) blocks with 6 of the familiarized NADs and 17 alternating (A) blocks with 3 familiarized NADs and 3 NAD violations presented in strictly alternating order (Fig. 2). NA and A blocks were presented as shown in Fig. 2 with each session starting with an NA block. During the test phase, stimuli were presented in blocks of 6 stimuli with a total block length of 18 s followed by an 18 s silent baseline period to allow the oxygenation levels to return to baseline. Inter-stimulus intervals in both the familiarization and the test phase randomly varied between 200 and 400 ms for the non-linguistic experiment and varied within this range depending on the length of the sentences for the linguistic experiment. Four pseudo-randomized stimulus lists were constructed from the stimulus set and counterbalanced between participants. Each list contained the stimuli for both the familiarization and test phase, with no repetition of individual stimuli. Each of the NADs (*sta x-ando*/*puo x-are* or *sta x-are/puo x-ando*) occurred a maximum of 3 times in a row in both the familiarization and test phase. In line with earlier studies using such an fNIRS paradigm (Gervain et al., 2012), we expected enhanced neural activation in response to *alternating* (A) compared to *non-alternating* (NA) blocks, if NAD violations were perceived as being different from the familiarized NADs. Although it is possible that further learning occurs during the test phase, our approach comparing all *A* blocks to all *NA* blocks primarily captures NAD discrimination after learning, rather than the learning process itself.

**Fig. 3:**
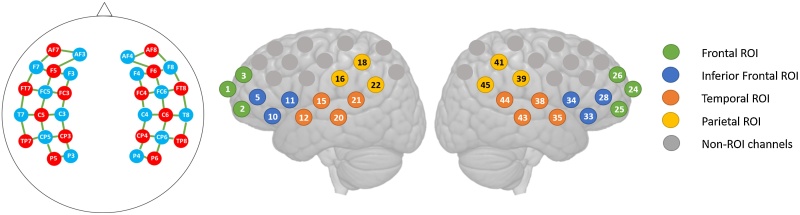
fNIRS optode layout. Top left: Positions of fNIRS sources (red) and detectors (blue) relative to the 10-10 system. Top right: color-coded Regions of Interest (ROIs). Bottom: NIRS channels on the MNI ICBM-152 template implemented in NIRSLab. ROIs: green: frontal, blue: inferior frontal, orange: temporal, yellow: parietal, grey: channels not included in the ROIs.

FNIRS data were recorded using a NIRx NIRScout 16 × 16 continuous wavelength system with NIRStar acquisition software (Version 15.0, NIRx Medical Technologies, Berlin, Germany) with 16 LED sources (wavelengths: 760 and 850 nm) and 16 photodetectors. All optodes were arranged in a flexible cap (EASYCAP GmbH, Woerthsee-Etterschlag, Germany) to create 46 measurement channels covering bilateral frontal, temporal and parietal brain regions (Source-Detector distance: 2.5 cm, see Fig. 3). Regions of interest (ROIs) included the bilateral temporal, parietal, inferior frontal and prefrontal cortex. Data were acquired with a sampling rate of 6.25 Hz. During data acquisition, the participating child sat in a car seat in a sound-attenuated booth. The NIRS cap was fitted before starting the familiarization. It was positioned relative to the ears and the midline such that the optode positions corresponded to the positions of the international 10−10 system, shown in Fig. 3. Since the cap was fitted purely based on skull landmarks and no individual anatomical brain data were available, individual channel positions should be interpreted with caution and for data analysis, data from single channels were pooled in spatial ROIs (see Fig. 3). Optode cables were fixed by cap-mounted cable holders and relieved by a cable arm, reducing optode movement. A small video camera was used to observe the child during the experiment.

Stimulus presentation started after calibration, performed to optimize the NIRS signal. Stimuli were played at approximately 72 dB SPL (at the child’s position) via two loudspeakers facing the child. To minimize head movement, silent cartoons were played on a 14” laptop screen facing the child at approximately 50 cm throughout the familiarization and test phases. The accompanying caregiver was seated next to the child and was instructed not to speak to the child during the experiment. In total, stimulus presentation lasted a maximum of 25 min, but was stopped earlier if the child became fussy, the caregiver requested so, or the NIRS cap had been displaced.

### 2.4 Data pre-processing

Data pre-processing and block averaging were performed in NIRSlab v. 2016.01 (NIRx Medical Technologies, Berlin, Germany). First, the quality of the raw attenuation data was assessed for each participant by means of visual inspection. Blocks containing movement artifacts were excluded from further analysis. Only datasets with a minimum of four remaining blocks per block type (A and NA) were included. The retained data set included a mean of 8.9 alternating blocks (SD: 2.9) and 9.3 non-alternating blocks (SD: 3.0) per participant, with no significant difference in number of trials retained per condition (t = 1.8, p = 0.07). Means and ranges of included trials per age group and experiment are shown in Table A1 of the Appendix. Because children get bored and start to move after prolonged testing, the number of infants contributing data decreased for both NA and A blocks toward the end of the test phase. No other clustering of retained blocks was found in the data.

Attenuation data were subsequently filtered using a band-pass filter with a low cut-off of 0.01 Hz and a high cut-off of 0.7 Hz and a roll off width of 15 %. HbO (oxygenated hemoglobin) and HbR (de-oxygenated hemoglobin) concentration changes were then computed from the attenuation data using the modified Beer-Lambert law with the extinction coefficients by W.B. Gratzer and N. Kollias, implemented in NIRSlab. Concentration changes were computed assuming the same Differential Pathlength Factor (DPF) for both ages, sexes and all channels. To account for potential differences in DPF, we only report on differences between conditions in HbO and HbR concentration changes, respectively. Since optodes were placed in positions from the international 10−10 system, source-detector distances differed slightly between channels. The MNI ICBM-152 head model implemented in NIRSlab (Mazziotta et al., 2001) was used to compute S-D distances based on the S-D distance of the first channel (2.5 cm for both ages), which were then considered when computing HbO and HbR concentration changes.

Individual blocks were baseline-corrected by subtracting the mean HbO and HbR amplitude during the silence period 1 s prior to stimulation from the HbO and HbR amplitudes of the stimulation block, respectively. Subsequently, included blocks were averaged for each participant per hemoglobin type (HbO and HbR), channel and block type (A and NA) to create block averages. Based on the timing of the hemodynamic response, mean HbO and HbR changes over the time window between 5−25 s past stimulus onset were extracted for each participant and channel for further statistical analysis.

### 2.5 Statistical analysis

Separate linear mixed-effect models were calculated for HbO and HbR effects in the linguistic and the non-linguistic experiments using *lme4* (Bates et al., 2014) in R Studio Version 1.1.453 (RStudio Team, 2016). Children familiarized with the incorrect NADs did not differ from children familiarized with the correct NADs in their HbO and HbR responses in the A and NA condition in either experiment. Similarly, no differences were found between the responses of children who participated in both experiments and children who participated in only one experiment, nor were any differences found related to the order in which children participated in the experiments. Data from all of these groups were therefore collapsed. Fixed effects of *Condition* (A and NA)*, Age* (2 and 3 years) and *ROI* (left- and right-hemispheric frontal, inferior frontal, temporal, and parietal regions, see Fig. 3 for channels included in each ROI) (with interaction term) as well as a random intercept for *Participant* were entered into the model. A sum contrast was used for the factor *Region* in order to compare each region to the grand mean. Based on our hypothesis that sensitivity to NAD violations (i.e., effect of *Condition*) might change between 2 and 3 years of age and would most likely be concentrated in left-hemispheric language-related regions, a full model including a 3-way interaction between the fixed effects was compared with a reduced model specifying only main effects using a likelihood ratio test.

Since HbO and HbR responses are anti-correlated in a typical hemodynamic response and infant HbO concentration changes are more sensitive than HbR concentration changes (e.g., Gervain et al., 2008), we expected opposite effects in HbO and HbR responses with larger effects in HbO data. Linear mixed effects analyses were followed up with pair-wise comparisons for the effect of *Condition* for each *Age* and *ROI*, corrected for multiple comparisons using Tukey’s method.

## 3 Results

### 3.1 NAD processing in the linguistic domain

The Linear Mixed Effects analysis for the *linguistic* experiment revealed a statistically significant 3-way interaction between the effects of *Condition, Age* and *ROI* on HbO and HbR concentration changes (HbO: χ^2^ = 34.9, *p* = 0.04; HbR: χ^2^ = 42.9, *p* = 0.005). These results suggest that activation differences between *Alternating* and *Non-Alternating* blocks in the test phase are modulated by age and ROI in the linguistic domain.

**Fig. 4:**
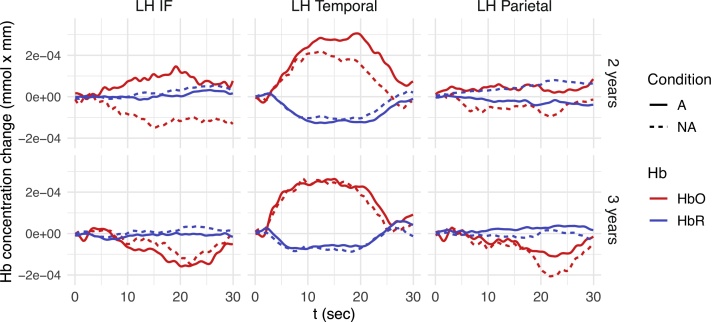
Mean time courses of oxygenated (red) and de-oxygenated (blue) hemoglobin for ROIs showing a significant A-NA difference in the linguistic experiment (top: 2-year-olds, bottom: 3-year-olds). The x-axis represents time where stimulation starts at 0 and lasts 18 s. Solid lines: alternating blocks (A), dotted lines: non-alternating blocks (NA), LH: left hemisphere, IF: inferior frontal ROI. HbO responses to A vs. NA blocks differ significantly in the left Temporal, IF and parietal ROI in 2-year-old children.

Planned comparisons (Tukey’s method) for the effect of *Condition* for each *Age* and *ROI* for the linguistic experiment (shown in Table 1) revealed significantly larger HbO concentration changes for A blocks compared to NA blocks (indicating the detection of NAD violations) in left-hemispheric inferior frontal, temporal, and parietal regions at 2 years, but no significant HbO concentration changes at 3 years of age (see Fig. 4). HbR data corroborated these results, with significant A vs. NA concentration differences found in the left-hemispheric parietal region (Appendix, Table A2) in 2-year-olds, but no significant concentration changes in 3-year-olds. Note that the hemodynamic responses in the left inferior frontal and parietal regions are inverted (i.e., show negative HbO and positive HbR responses) for both conditions in 3-year-olds and for NA only in 2-year-olds. The negative t-values for HbR, presented in the Appendix, reflect effects in the same direction as found in HbO data, since HbR concentration changes are negatively related to HbO concentration changes. Thus, our results indicate that 2-year-old, but not 3-year-old toddlers are able to detect linguistic NAD violations during passive listening and that left-hemispheric inferior frontal, temporal, and parietal regions are involved in the detection of these violations.

**Table 1:** Planned comparisons (Tukey) of Alternating (A) vs. Non-alternating (NA) blocks for each age group and ROI in the linguistic domain (HbO). Statistically significant effects are marked in **bold**.

Contrast	Age	Region	Estimate (*10^−5^)	SE (*10^−5^)	df	t-ratio	*p*-value
A - NA	2	LH Frontal	7.23	4.89	2550	1.48	0.1391
**A - NA**	**2**	**LH IF**	**18.43**	**4.92**	**2550**	**3.745**	**0.0002**
**A - NA**	**2**	**LH Temporal**	**8.49**	**4.21**	**2550**	**2.018**	**0.0437**
**A - NA**	**2**	**LH Parietal**	**9.96**	**4.89**	**2550**	**2.036**	**0.0418**
A - NA	2	RH Frontal	8.16	4.86	2550	1.68	0.093
A - NA	2	RH IF	−6.30	4.89	2550	−1.289	0.1975
A - NA	2	RH Temporal	2.88	4.25	2550	0.678	0.498
A - NA	2	RH Parietal	5.86	4.96	2550	1.182	0.2373
A - NA	3	LH Frontal	−4.37	4.86	2550	−0.9	0.3683
A - NA	3	LH IF	−3.44	4.86	2550	−0.708	0.4792
A - NA	3	LH Temporal	0.15	4.21	2550	0.353	0.724
A - NA	3	LH Parietal	3.81	4.89	2550	0.78	0.4358
A - NA	3	RH Frontal	2.92	4.89	2550	0.597	0.5507
A - NA	3	RH IF	−3.42	4.92	2550	−0.695	0.4871
A - NA	3	RH Temporal	4.98	4.23	2550	1.179	0.2387
A - NA	3	RH Parietal	0.24	4.86	2550	0.049	0.9607

### 3.2 NAD processing in the non-linguistic domain

The Linear Mixed Effects analysis for the *non-linguistic* experiment revealed a statistically significant interaction between *Condition* and *Age* on HbO concentration changes (χ^2^ = 14.6, *p* = 0.0001), as well as a main effect of *ROI*. An additional model containing a 3-way interaction (Condition*Age*ROI) was tested, but the likelihood ratio test did not reach significance (χ^2^ = 31.0, *p* = 0.096). Planned comparisons (Tukey’s method) for the effect of *Condition* for each *Age* showed significantly larger HbO concentration changes for A compared to NA blocks in 3-year-olds, but not in 2-year-olds (Table 2). This result indicates that 3-year-old children were able to detect NAD violations in tone sequences, while 2-year-olds were not. Mirroring the analysis of the linguistic experiment, we performed planned comparisons per ROI for the 3-year-olds, which showed significant condition differences in bilateral temporal and parietal ROIs (Table 2). For HbR concentration changes, a significant Condition*Age*ROI interaction (χ^2^ = 36.6, p = 0.026) was found, where HbR responses to A blocks were larger than those to NA blocks in the left temporal and parietal ROI, while NA blocks showed a larger HbR response than A blocks in the right frontal ROI (Appendix, Table A3). This direction difference is characterized by an inverted response to the NA condition in the right frontal ROI. Inverted hemodynamic responses to NA blocks were also found in bilateral parietal regions at 3 years of age. Both for HbO and HbR, significant A vs. NA differences were found in 3-year-olds only. These data show that 3-year-olds, but not 2-year-olds are able to learn NADs in the non-linguistic experiment.

**Table 2:** Planned comparisons (Tukey) of Alternating (A) vs. Non-alternating (NA) blocks for each age group and ROI in the non-linguistic domain (HbO in mmol x mm). Statistically significant effects are marked in **bold**.

Contrast	Age	Region	Estimate (*10^−5^)	SE (*10^−5^)	df	t-ratio	p-value
A - NA	2 years	LH Frontal	4.36	3.73	2534	1.171	0.2418
A - NA	2 years	LH IF	−0.69	3.78	2534	−0.184	0.8543
A - NA	2 years	LH Temporal	−5.49	3.24	2534	−1.693	0.0907
A - NA	2 years	LH Parietal	−2.38	3.73	2534	−0.638	0.5237
A - NA	2 years	RH Frontal	6.26	3.75	2534	1.668	0.0954
A - NA	2 years	RH IF	−2.67	3.80	2534	−0.701	0.4831
A - NA	2 years	RH Temporal	3.5	3.24	2534	1.079	0.2808
A - NA	2 years	RH Parietal	−2.50	3.78	2534	−0.663	0.5076
A - NA	3 years	LH Frontal	6.49	3.78	2534	1.718	0.0859
A - NA	3 years	LH IF	5.61	3.83	2534	1.464	0.1433
**A - NA**	**3 years**	**LH Temporal**	**7.45**	**3.24**	**2534**	**2.297**	**0.0217**
**A - NA**	**3 years**	**LH Parietal**	**7.82**	**3.78**	**2534**	**2.07**	**0.0386**
A - NA	3 years	RH Frontal	7.03	3.73	2534	1.887	0.0592
A - NA	3 years	RH IF	4.68	3.73	2534	1.256	0.2093
**A - NA**	**3 years**	**RH Temporal**	**7.83**	**3.26**	**2534**	**2.403**	**0.0163**
**A - NA**	**3 years**	**RH Parietal**	**7.60**	**3.78**	**2534**	**2.013**	**0.0442**

**Fig. 5:**
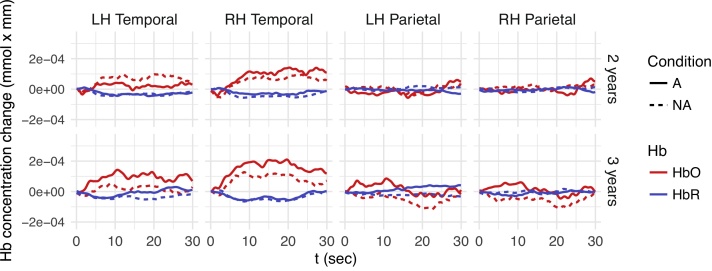
Mean time courses of oxygenated (red) and de-oxygenated (blue) hemoglobin per ROI for the non-linguistic experiment (top: 2-year-olds, bottom: 3-year-olds). The x-axis represents time where stimulation starts at 0 and lasts 18 s. Solid lines: alternating blocks (A), dotted lines: non-alternating blocks (NA), LH: left hemisphere, RH: right hemisphere. HbO responses to A vs. NA blocks differ significantly 3-year-old, but not in 2-year-old children.

Fig. 5 shows fNIRS responses for ROIs for which the A vs. NA differences were most pronounced in the non-linguistic experiment. Note that this figure also shows smaller HbO responses to non-linguistic stimuli than were found for linguistic stimuli in the temporal ROI (compare Fig. 4). This was confirmed by a comparison over all eight ROIs including both *A* and *NA* blocks, which revealed that HbO responses to non-linguistic stimuli were significantly smaller than HbO responses to linguistic stimuli (Welch’s t = 3.89, p = 0.0001).

To explore how NAD learning in the non-linguistic domain relates to NAD learning in the linguistic domain Pearson’s correlation analyses were performed for the subset of children who provided sufficient quality data in both tasks (2 years: N = 17, 3 years: N = 15). This analysis revealed no significant relation between NAD discrimination (A vs. NA difference) in the linguistic and the non-linguistic domains after correction for multiple comparisons. Before correction, a significant (r = .488, p = 0.047) correlation was observed between left temporal fNIRS responses to alternating blocks in the linguistic and non-linguistic domain, but this effect did not survive correction for multiple comparisons.

## 4 Discussion

The aim of the present study was twofold. First, we aimed to study the brain regions underlying the neural mechanisms supporting NAD learning from passive listening under the hypothesis that the neural correlates of NAD learning under passive listening undergo a developmental shift between the ages of 2 and 4 years (Mueller et al., 2019). Secondly, we aimed to investigate to what extent NAD learning abilities in early childhood are rooted in domain-general learning mechanisms by using the same paradigm to test children’s ability to learn NADs from sentences and tone sequences. To our knowledge, this study is the first to directly compare NAD learning in the linguistic and the non-linguistic domain at this age.

Our data showed neural evidence of linguistic NAD learning in 2-year-old, but not in 3-year-old children, which might be explained by a developmental shift in the mechanisms for NAD learning in the linguistic domain (as previously suggested by Skeide and Friederici, 2016), taking place during the third year of life. The absence of neural detection of NAD violations after passive listening in 3-year-old children is in line with a recent ERP study, which found no ERP evidence of NAD violation detection on the group level in 4-year-olds (Mueller et al., 2019). Since no differences were found between children familiarized with correct and children familiarized with incorrect Italian sentences, 2-year-olds’ responses to violations cannot be explained by previous knowledge of Italian or by specific combinations between auxiliary/modal verb and verb suffix. Therefore, NAD learning must have occurred during the familiarization phase^1^ of the experiment. Our findings thus corroborate and extend recent results showing a developmental shift in the neural signature for NAD learning from syllable triplets between the ages of 2 and 4 years (Mueller et al., 2019) and might further specify the time window in which developmental changes in NAD learning under passive listening take place. The present study used a passive listening paradigm to tap into associative NAD learning abilities. Although it is possible that abstract reasoning in combination with native-language knowledge might contribute to learning under passive listening at these ages (e.g., Téglás et al., 2011; Tenenbaum et al., 2011), this learning route is expected to become more rather than less accessible between the ages of 2 and 3 years, as native language knowledge further develops. In order to confirm whether older children and adults show more controlled learning of these NADs and to reveal the brain regions involved, further studies using active paradigms are needed.

The detection of linguistic NAD violations after familiarization through passive listening in 2-year-old children was shown to be subserved by left-hemispheric inferior frontal, temporal and parietal regions. The involvement of the left temporal cortex in 2-year-olds is in line with the hypothesis that NAD learning in early development is driven by associative learning processes rooted in the temporal cortex (Friederici et al., 2011; Skeide and Friederici, 2016) as well as with previous fNIRS data showing involvement of the temporal cortex in detecting syllable repetition in trisyllabic pseudo-words at birth (Gervain et al., 2008). Since our paradigm measures the detection of violations, it cannot confirm whether the temporal cortex was also involved in the learning process itself. The additional involvement of the left inferior frontal region in 2-year-olds’ linguistic NAD processing is in line with the adult literature (e.g., Folia and Petersson, 2014; Friederici, 2018; Uddén et al., 2017) on the detection of syntactic rule violations. Moreover, this region was also involved in the detection of syllable repetitions in newborns (Gervain et al., 2008, 2012), suggesting an early role in processing speech structure. Our findings extend previous work by showing that at 2 years of age, as in adults, the inferior frontal region is involved in the detection of violations of NADs in natural language and that this effect is left-lateralized, even when these NADs are acquired through passive listening. Since the dorsal pathway targeting the inferior frontal gyrus in adults is not yet myelinated at birth, the sensitivity of the left inferior frontal cortex to violations of learned linguistic structure in infants and younger children might be supported by the second dorsal pathway connecting the left superior temporal cortex to the premotor cortex (Perani et al., 2011; Skeide and Friederici, 2016). Finally, the left parietal region showed evidence of detection of NAD violations. The observed difference in HbO concentration in the left parietal region should be interpreted with caution, since the alternating condition does not induce a typical hemodynamic response, but a rather flat response. This result therefore contributes to the overall picture showing detection of linguistic NAD violations in the left-hemispheric language network, but cannot be interpreted individually.

In the non-linguistic domain, detection of NAD violations was found at 3 years, but not at 2 years of age and was shown to be subserved by a bilateral brain network. Although recent data suggest that younger infants are able to learn NADs in tone sequences (Winkler et al., 2018), these NADs were marked by non-adjacent repetitions of identical tones, where the identity relation might have provided a crucial cue (see Gebhart et al., 2009). The present study is amongst the first to study non-linguistic NAD learning in early childhood using NADs between non-identical tones, for which processing cannot be explained by repetition detection and is thus more comparable to NADs found in natural language. While our data show a decrease in the neural responses related to linguistic NAD learning with age, an increase was observed for non-linguistic NAD learning, suggesting that the neural underpinnings of non-linguistic NAD learning under passive listening develops years after the ability to acquire linguistic NADs under the same conditions (Friederici et al., 2011). This finding provides evidence against the idea that linguistic NAD learning abilities might be rooted in a domain-general learning mechanism.

The anatomical distribution of the fNIRS responses related to NAD violation detection in non-linguistic material also differs from that for linguistic stimuli, pointing toward domain-specific learning mechanisms for linguistic NADs. While core left-hemispheric regions in the language network are involved in the detection of linguistic NAD violations, the absence of an interaction with *ROI* in the non-linguistic experiment indicates that the effect here was more bilaterally wide-spread. In fact, when comparing individual ROIs with their contralateral homologue, no lateralization was found for the sensitivity to non-linguistic NAD violations. These results are in line with previous findings in adults (Cheung et al., 2018), which showed that detection of NAD violations in musical motifs activates the right inferior frontal gyrus and bilateral insulae in trained musicians. Similarities in the response patterns in the linguistic and non-linguistic domain include the presence of inverted hemodynamic responses (i.e., where HbO decreases and HbR increases). Whereas temporal responses were canonical in shape when processing A and NA blocks of speech and tone sequences, (inferior) frontal and parietal regions showed inverted responses in both the linguistic and the non-linguistic domain. Specifically, in the linguistic domain, 2-year-old children show inverted responses to NA blocks, while showing positive HbO and negative HbR changes to A blocks (containing violations) in the left inferior frontal region. Since inverted responses are not generally expected for auditory or linguistic stimuli, these might be an effect of repetition suppression caused by habituation during the familiarization phase (Issard and Gervain, 2018), which is only followed by dishabituation if violations are detected. Furthermore, since some of the regions involved in violation detection in linguistic and non-linguistic NADs overlap, we cannot exclude the possibility that violation detection after NAD learning partly depends on domain-general mechanisms, as was proposed for statistical learning of adjacent dependencies (Frost et al., 2015). However, our data do not suggest a shared neural basis for linguistic and non-linguistic NAD learning in early development. Regarding the domain-generality of the learning mechanisms underlying NAD learning, our results thus paint a different picture than the adult literature. As described in the introduction, adults learn non-linguistic NADs under similar circumstances as linguistic NADs (Creel et al., 2004; Endress, 2010; Gebhart et al., 2009; Pacton et al., 2015; Pacton and Perruchet, 2008) and recruit the left inferior frontal cortex to detect violations in linguistic NADs (Bahlmann et al., 2008; Friederici et al., 2006, 2013) and its contralateral homologue to detect NAD violations in music (Cheung et al., 2018). In contrast, our results show that these abilities both follow distinct developmental trajectories and show a different neural basis in early development.

A possible caveat in the comparison between the linguistic and non-linguistic domain in this study is posed by the differences in complexity between the two stimulus sets: the non-linguistic stimuli consist of pure tones, while each syllable in the linguistic stimuli carries higher-level spectral and temporal information. The effect of increasing cognitive control (Skeide et al., 2016) and endogenous attention (de Diego-Balaguer et al., 2016; Martinez-Alvarez et al., 2017) with age might interact with stimulus complexity, leading to different developmental trajectories. Indeed, stimulus complexity was previously shown to influence the hemodynamic response in infants, where overall smaller hemodynamic responses to non-linguistic NADs might be an indication of reduced or more superficial processing (see Issard and Gervain, 2018). However, our use of pure tones for the non-linguistic stimuli was motivated by our aim to distinguish between linguistic and non-linguistic processing. Phonological information (Azadpour and Balaban, 2008) and native language knowledge (Berent et al., 2010) were shown to influence the processing of complex non-linguistic sounds. Therefore, using acoustically complex non-linguistic stimuli would not have allowed us to adequately distinguish language-specific from domain-general NAD learning mechanisms. Furthermore, although differences in hemodynamic responses to the linguistic and non-linguistic stimuli might be attributed to stimulus complexity, this is unlikely to be the case for differences between responses to familiarized NADs and NAD violations within one domain. It is therefore unlikely that the differences in NAD learning found between the linguistic and non-linguistic domain are due to differences in stimulus complexity.

## 5 Conclusion

In conclusion, our study provides new evidence supporting the notion of a developmental shift in NAD learning in the linguistic domain, taking place during the third year of life. The present data show violation detection in NADs learned under passive listening in 2-year-old, but not in 3-year-old children, that is sub-served by a left-hemispheric language network. Moreover, NAD learning in the non-linguistic domain was only observed at the age of 3 years and NAD violation detection involved a bilateral network including frontal, temporal, and parietal regions. Thus, NAD learning undergoes different developmental changes and depends on different brain regions in the linguistic and non-linguistic domains, suggesting that these NAD learning abilities might be rooted in distinct learning mechanisms during development.

## Funding

This work was funded by the 10.13039/501100001659Deutsche Forschungsgemeinschaft (DFG, German Research Foundation) in the Research Unit FOR 2253: Crossing the borders, Project 1 (WA2969/6-1; HO1960/18-1; FR 519/20-1; Project number 258522519).

## Data statement

Due to the sensitive nature of the data obtained from children, parents were assured that raw data would remain confidential and would not be shared.

## Declaration of Competing Interest

None.

## Figures and Tables

**Table A1:** Means and ranges for the number of included trials per condition, age group and domain.

Domain	Age	Mean NA	Mean A	Range NA	Range A
Linguistic	2 years	9.0	8.68	5−15	5−16
Linguistic	3 years	9.12	8.84	4−15	4−15
Non-linguistic	2 years	9.12	9.2	5−16	4−16
Non-linguistic	3 years	9.92	8.56	5−14	5−13

**Table A2:** Planned comparisons (Tukey) of Alternating (A) vs. Non-alternating (NA) blocks for each age group and ROI in the linguistic domain (HbR in mmol x mm). Statistically significant effects are marked in **bold**.

Contrast	Age	Region	Estimate (*10^−5^)	SE (*10^−5^)	df	t-ratio	*p*-value
A - NA	2 years	LH Frontal	−3.08	2.11	2550	−1.463	0.1435
A - NA	2 years	LH IF	−1.48	2.12	2550	−0.700	0.4842
A - NA	2 years	LH Temporal	−1.76	1.81	2550	−0.970	0.3325
**A - NA**	**2 years**	**LH Parietal**	**−6.83**	**2.11**	**2550**	**−3.245**	**0.0012**
A - NA	2 years	RH Frontal	−1.30	2.09	2550	−0.622	0.5343
A - NA	2 years	RH IF	−0.68	2.11	2550	−0.321	0.7483
A - NA	2 years	RH Temporal	−2.58	1.83	2550	−1.413	0.1578
A - NA	2 years	RH Parietal	−2.10	2.13	2550	−0.984	0.3254
A - NA	3 years	LH Frontal	2.41	2.09	2550	1.152	0.2495
A - NA	3 years	LH IF	−2.03	2.09	2550	−0.968	0.3329
A - NA	3 years	LH Temporal	0.93	1.81	2550	0.515	0.6068
A - NA	3 years	LH Parietal	2.77	2.11	2550	1.314	0.1889
A - NA	3 years	RH Frontal	−0.82	2.11	2550	−0.389	0.6976
A - NA	3 years	RH IF	−1.62	2.12	2550	−0.763	0.4455
A - NA	3 years	RH Temporal	1.84	1.82	2550	1.008	0.3134
A - NA	3 years	RH Parietal	2.04	2.09	2550	0.974	0.3301

**Table A3:** Planned comparisons (Tukey) of Alternating (A) vs. Non-alternating (NA) blocks for each age group and ROI in the non-linguistic domain (HbR in mmol x mm). Statistically significant effects are marked in **bold**.

Contrast	Age	Region	Estimate (*10^−5^)	SE (*10^−5^)	df	t-ratio	*p*-value
A - NA	2 years	LH Frontal	−3.02	1.54	2534	−1.957	0.0504
A - NA	2 years	LH IF	−1.12	1.57	2534	−0.716	0.4744
A - NA	2 years	LH Temporal	0.23	1.34	2534	0.17	0.865
A - NA	2 years	LH Parietal	−1.11	1.54	2534	−0.718	0.4731
A - NA	2 years	RH Frontal	−0.006	1.55	2534	−0.004	0.9968
A - NA	2 years	RH IF	0.87	1.58	2534	0.551	0.5818
A - NA	2 years	RH Temporal	1.96	1.34	2534	1.457	0.1452
A - NA	2 years	RH Parietal	0.63	1.57	2534	0.404	0.6864
A - NA	3 years	LH Frontal	−2.14	1.57	2534	−1.368	0.1716
A - NA	3 years	LH IF	−0.13	1.59	2534	−0.081	0.9352
**A - NA**	**3 years**	**LH Temporal**	**3.06**	**1.34**	**2534**	**2.275**	**0.023**
**A - NA**	**3 years**	**LH Parietal**	**3.85**	**1.57**	**2534**	**2.458**	**0.014**
**A - NA**	**3 years**	**RH Frontal**	**−4.74**	**1.54**	**2534**	**−3.067**	**0.0022**
A - NA	3 years	RH IF	−1.74	1.54	2534	−1.13	0.2588
A - NA	3 years	RH Temporal	0.81	1.35	2534	0.596	0.551
A - NA	3 years	RH Parietal	−0.88	1.57	2534	−0.561	0.5752

## Appendix A

Table A1

## Footnotes

1 There was no statistically significant change in the size of the learning effect (A-NA) in either age group when comparing the first half of the test phase to the second half. However, these results have to be taken with caution because the number of trials in the second half of the experiment is significantly smaller than in the first half. The full analysis is made available on Open Science Framework under the following link: https://osf.io/4ywhe/.
